# From bug to bubble: the rising prominence of *Lactobacillus*-derived extracellular vesicles as postbiotic wound healers

**DOI:** 10.20517/evcna.2026.59

**Published:** 2026-07-29

**Authors:** Yuting Li, Min Zhang, Yirong Wang, Xiangyu Sang, Wenchen Cai, Yejiao Shi, Honggang Hu

**Affiliations:** ^1^Institute of Translational Medicine, Shanghai University, Shanghai 200444, China.; ^2^School of Medicine, Shanghai University, Shanghai 200444, China.; ^3^Faculty of Chinese Medicine, Macau University of Science and Technology, Macau 999078, China.; ^4^MedEng-X Institutes, Shanghai University, Shanghai 200444, China.; ^5^Institute of Translational Medicine, Shanghai Jiao Tong University, Shanghai 200030, China.; ^6^School of Pharmacy, Chengdu Medical College, Chengdu 610083, Sichuan, China.; ^#^These authors contributed equally to the work.

**Keywords:** Bacterial extracellular vesicles, wound healing, gut-skin axis, probiotics, *Lactobacillus*

## Abstract

The therapeutic landscape of probiotics is undergoing a paradigm shift from the live bacteria “bug” to the bioactive extracellular vesicles (EVs) “bubble” they secrete. Owing to their essential defense and metabolic functions, EVs derived from *Lactobacillus*, a genus of anaerobic or microaerophilic probiotics well known for their ability to ferment carbohydrates into lactic acid, have garnered particular interest for wound healing. As such, the presented mini-review began by elucidating why these *Lactobacillus-*derived EVs (L-EVs) attract upward attention by highlighting their superior safety, stability, multifunctionality, customizability, and scalability. This was followed by elaborating how these L-EVs accelerate wound healing by detailing their multifaceted mechanisms, including removing bacteria, reprogramming the microenvironment, repairing tissue, and remodeling the matrix during the wound-healing process. Lastly, it ended by envisioning where these L-EVs head for future therapy by addressing the translational hurdles, such as the lack of isolation and purification standardizations, the existing cargo and batch variabilities, as well as the ambiguous classification and regulation of L-EVs, which must be overcome to push them from bench to bedside. Moreover, it further proposed a specialized database based on artificial intelligence and multi-omics, the combined modification based on diverse engineering approaches, as well as the advanced formulation based on the material engineering strategies of L-EVs in the future to integrate the next generation of wound management modalities with optimal treatment efficacy.

## WHY THE LACTOBACILLUS DERIVED EXTRACELLULAR VESICLES ATTRACT UPWARD ATTENTION

*Lactobacillus* is a genus of anaerobic or microaerophilic gram-positive bacteria with a rod shape^[1]^. These bacteria are the primary members of the lactic acid bacteria (LAB) group and are known for their ability to ferment carbohydrates into lactic acid^[2]^. They naturally exist in the human body and are important components of the microbiome in the gut and vagina^[3-6]^. By producing lactic acid and other short-chain fatty acids (SCFAs), these *Lactobacillus* species provide an acid mantle with a pH typically between 4.5 and 5.5 for the host, inhibiting the overgrowth of opportunistic pathogens such as *Staphylococcus aureus* and *Escherichia coli*, while facilitating the absorption of certain nutrients such as calcium and iron^[7,8]^. Besides, they are also capable of protecting the host against pathogen invasion by competing for adhesion sites and nutrients on epithelial surfaces, as well as by producing antimicrobial peptides and bacteriocins^[9,10]^. More importantly, they can interact with the toll-like receptors on immune cells as well, promoting immune tolerance to commensal microbes while priming defenses against actual threats^[11]^.

Owing to these essential defense and metabolic functions, the *Lactobacillus* species are widely classified as probiotics and exploited as live biotherapeutics for the treatment of gastrointestinal disorders as well as vaginal infections^[12]^. However, even though being considered safe for healthy individuals, the live probiotics still pose risks to vulnerable populations^[13]^. Cases of severe infections have been reported in premature infants and immunocompromised hospital patients who were administered the live probiotics^[14,15]^. Furthermore, maintaining the viability of the *Lactobacillus* at the disease site is also technically challenging. The hostile environment of the disease site, typically characterized by high protease activities and shifted pH levels, may compromise the survival and functional activities of the *Lactobacillus*, leading to inconsistent therapeutic outcomes^[16]^. These limitations expedited the development of the *Lactobacillus-derived* extracellular vesicles (L-EVs), which are nonviable nanostructures containing microbial components or metabolites that confer health benefits to the host with an improved safety profile^[17]^.

The surge of interest in L-EVs is not merely a trend but a response to their unique properties. First of all, as cell-free entities, the L-EVs do not replicate and cannot transfer pathogenic genes in the same manner as live bacteria. Therefore, they eliminate the risk of systemic infection and sepsis. Secondly, their lipid bilayer nanostructures provide inherent protection for their bioactive cargoes, ensuring the stability and potency of these cargoes in complex bodily fluids^[18,19]^. Their nanostructures also make them ideal for incorporation into various formulations, including injections, sprays, and gels. Thirdly, the L-EVs inherit the multifaceted biological molecules and activities of their parent *Lactobacillus* strains. Therefore, they are capable of exhibiting multifunctional effects, such as antimicrobial, anti-inflammatory, as well as regenerative effects simultaneously. Fourthly, the L-EVs are customizable according to the diverse therapeutic requirements. Based on biological, physical, as well as chemical engineering approaches, the cargo required can be either displayed to or loaded into the L-EVs to achieve the optimal therapeutic efficiency^[20]^. Last but not least, the L-EVs are more scalable compared to the mammalian cell-derived extracellular vesicles (EVs) since the *Lactobacillus* can grow rapidly in cost-effective fermentation media, allowing for mass production and standardized purification. All these superiorities have made the L-EVs the primary focus for both researchers and clinicians [Figure 1].

**Figure 1:**
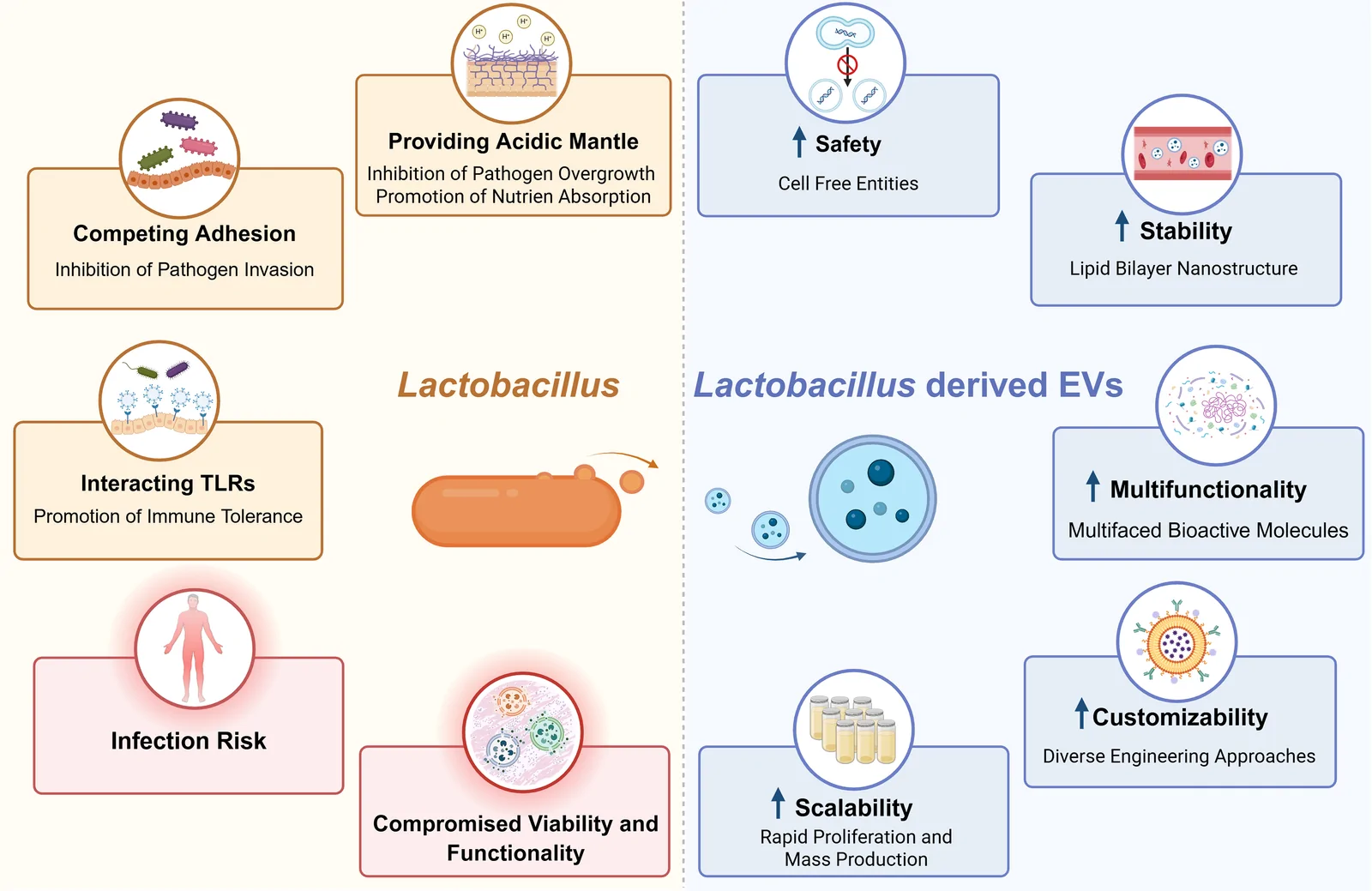
The advantages and shortcomings of *Lactobacillus* and L-EVs for wound management. Created in BioRender. Shi, Y. (2026). EV: Extracellular vesicle; L-EV: *Lactobacillus*-derived extracellular vesicle; TLR: Toll-like receptor.

## HOW THE LACTOBACILLUS DERIVED EVS ACCELERATE WOUND HEALING

The upward attention towards the L-EVs gradually revealed their antibacterial, anti-inflammatory, as well as regenerative capacities. Therefore, these L-EVs have been considered omnipotent candidates for wound management since the wound-healing process typically involves overlapping phases of hemostasis, inflammation, proliferation, and remodeling. By removing bacteria, reprogramming the microenvironment, repairing the tissue, and remodeling the wound, the L-EVs have been found to sequentially expedite the wound-healing process with outstanding performance [Figure 2 and Table 1].

**Figure 2:**
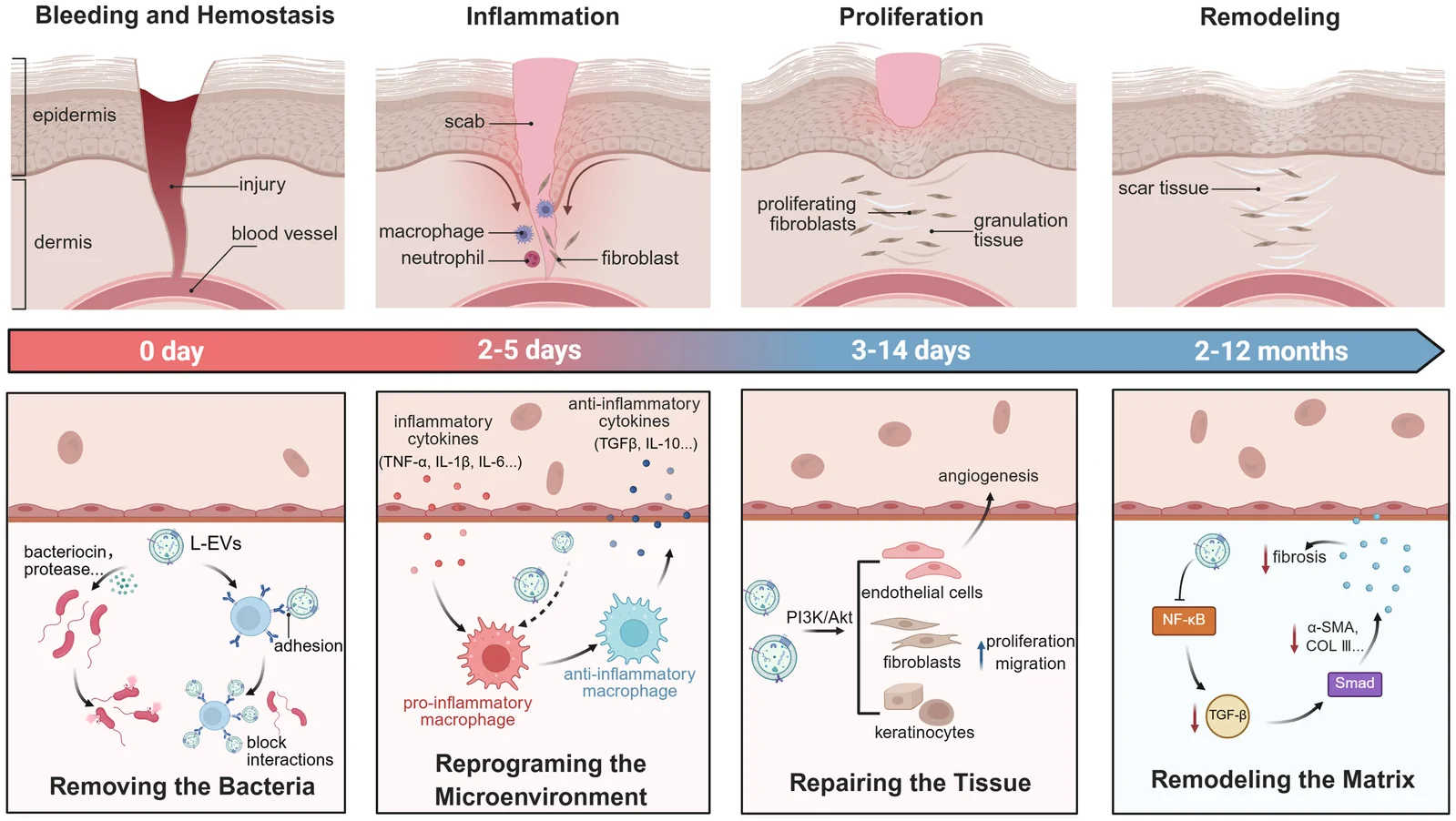
Illustration of the four phases during the wound healing process and the relevant functional mechanisms of L-EVs for accelerating wound repair and regeneration. The indicated time ranges are approximate estimates based on general wound-healing models. These phases are not strictly sequential and may overlap considerably depending on the wound type, size, and pathological conditions. Created in BioRender. Shi, Y. (2026). Akt: Protein kinase B; COL III: collagen type III; EV: extracellular vesicle; IL: interleukin; L-EV: *Lactobacillus*-derived extracellular vesicle; NF-κB: nuclear factor kappa B; PI3K: phosphoinositide 3-kinase; SMA: smooth muscle actin; TGF-β: transforming growth factor beta; TNF-α: tumor necrosis factor alpha.

**Table 1:** The L-EV in wound management: the biological activity, bioactive cargo, mechanistic pathway, animal model, and administration route

***Lactobacillus* strain**	**Biological activity**	**Bioactive cargo**	**Mechanistic pathway**	**Experimental model**	**Administration route**	**Ref.**
*Lactobacillus reuteri*	Anti-inflammation, promote M2 macrophage polarization, enhance keratinocyte migration, stimulate angiogenesis	Various bioactive metabolites	Nrf2-Keap1, NF-κB, PI3K/Akt, AMPK	Diabetic mouse model, *Staphylococcus aureus*-infected diabetic wound model	Topical administration	[27]
*Lactobacillus gasseri* BC12	Inhibit adhesion of opportunistic pathogens, enhance adhesion of beneficial lactobacilli	Adhesins: enolases, EF-Tu, chaperones	NR	NR	NR	[3]
*Lactobacillus reuteri*	Anti-inflammation, promote M2 macrophage polarization, improve collagen deposition, activate epithelial regeneration	3-HPA	NR	Tongue mucosal ulcer mouse model, full-thickness wound mouse model	Hydrogel-based topical administration	[26]
*Lactobacillus plantarum*	Anti-inflammation promote M2 macrophage polarization	NR	TLR2/4, NF-κB-related immune pathways	NR	NR	[30]
*Lactobacillus rhamnosus GG*	Enhance angiogenesis, boost proliferation and migration of keratinocytes, promote re-epithelialization, improve collagen deposition	miR-21-5p	PI3K-Akt, HIF1α	Full-thickness wound mouse model	Subcutaneous injection	[35]
*Lactobacillus plantarum*	Promote proliferation and migration of fibroblasts, increase HA synthesis	NR	Reverse UV-induced photoaging-related gene expression	Healthy adult volunteers	Topical administration	[36]
*Lactobacillus reuteri*	Promote proliferation and migration of keratinocytes, enhance angiogenesis, accelerate re-epithelialization	miR-21a-5p	PI3K-Akt	Diabetic mice model	Subcutaneous injection	[37]
*Lactobacillus casei, Lactobacillus plantarum*	Anti-inflammation, promote angiogenesis and re-epithelialization, reduce dermal thickness and scar risk	NR	NR	Full-thickness wound mouse model	Hydrogel-based topical administration	[38]
*Lactobacillus druckerii*	Promote angiogenesis and re-epithelialization, suppress dermal fibrosis, reduce collagen I/III and α-SMA expression	NR	MAPK, p-JNK/p-p38	Bleomycin-induced scleroderma mouse model, full thickness excisional burn wound mouse model	Subcutaneous injection	[40]

Akt: Protein kinase B; AMPK: AMP-activated protein kinase; EF-Tu: elongation factor Tu; EV: extracellular vesicle; HA: hyaluronic acid; HIF1α: hypoxia-inducible factor 1 alpha; L-EV: *Lactobacillus*-derived extracellular vesicle; MAPK: mitogen-activated protein kinase; miR: microRNA; NF-κB: nuclear factor kappa B; NR: not reported; Nrf2: nuclear factor erythroid 2-related factor 2; PI3K: phosphoinositide 3-kinase; SMA: smooth muscle actin; TLR: Toll-like receptor; UV: ultraviolet; 3-HPA: 3-hydroxypropionaldehyde; p-JNK: phosphorylated c-Jun N-terminal kinase.

### Removing the bacteria

While a balanced microbiome can support wound healing, the overgrowth of pathogenic bacteria or the formation of their biofilms creates significant barriers to tissue restoration. Therefore, removing the bacteria is the primary task of the inflammatory phase. At the beginning of the inflammatory phase, neutrophils are typically recruited to the wound site, undergo phagocytosis, and release reactive oxygen species (ROS) to eliminate bacteria^[21]^. Whereas in most cases, additional treatments such as debridement and antibiotics are still required in clinical settings. To more efficiently clear the persistent bacteria and terminate the chronic inflammation, more and more advanced treatment modalities, such as the AIE based photodynamic and photothermal therapies, have been developed recently, facilitating the transition from inflammation to proliferation^[22,23]^.

As most *Lactobacillus* have evolved antibacterial capacities to enhance competitive survival, the L-EVs have recently been found to exert antibacterial activities. These L-EVs with lipid bilayer nanostructures, on the one hand, can provide protection and enable delivery of their endogenous antibacterial substances for the direct bactericidal effects. For instance, the *Lactobacillus reuteri-*derived EVs contain the small molecular antibacterial agent reuterin, thus possessing bactericidal activity^[24-26]^. While their antibacterial activities have been preliminarily demonstrated by a series of pioneering studies, their therapeutic efficacy in managing more complex chronic wounds, such as infected diabetic wounds, remains limited. To facilitate effective chronic wound healing, additional antibacterial techniques or materials have been used in combination. For instance, Tai *et al.* reported a photothermal antibacterial system, which combined the *Lactobacillus reuteri*-derived EVs with indocyanine green (ICG) to effectively repair infected diabetic wounds^[27]^. On the other hand, these L-EVs can also act as adhesion analogs and block interactions between bacterial and host cells, thereby exerting indirect antibacterial effects. For instance, *Lactobacillus gasseri* BC12-derived EVs were capable of preventing pathogen attachment and supporting the colonization of beneficial species *in vitro*, suggesting that L-EVs may represent a candidate strategy in further investigation for wound healing applications^[3]^. Nevertheless, their *in vivo* therapeutic efficacy as adhesion analogs for the treatment of infected wounds is still waiting to be well evaluated.

### Reprogramming the microenvironment

With the waning of the neutrophils after approximately two days of the inflammatory phase, monocytes infiltrate the wound and differentiate into macrophages, which become the dominant cells from day 2 to day 5 after injury^[28]^. The macrophages are highly plastic and can transition from the pro-inflammatory M1 phenotype to the pro-regenerative M2 phenotype, initiating the next proliferation phase^[29]^.

Recent investigations have demonstrated that the L-EVs are uniquely suited to reprogram the immune macrophages and the inflammatory microenvironment of wounds. For instance, the previously mentioned *Lactobacillus reuteri-derived* EVs could also regulate inflammatory responses by modulating macrophage mitochondrial function through the inherent biomolecule 3-hydroxypropionaldehyde (3-HPA), promote macrophage M2 polarization, and ultimately facilitate wound healing^[26]^. While current study only investigated the regulatory effects of L-EVs on macrophages, without systematically examining their effects on keratinocytes, endothelial cells, or fibroblasts. Therefore, a complete mechanistic understanding of the cell network involved in wound repair has not been established. Besides, the *Lactobacillus plantarum-derived* EVs could induce the differentiation of human monocytic THP1 cells towards M2 macrophage polarization, downregulate inflammation-induced expression of M1 macrophage cell-surface markers, and exhibit an anti-inflammatory effect in the human skin *in vitro*^[30]^. However, it is worth noting that pro-inflammatory *vs*. anti-inflammatory effects vary across the different phases of wound healing. As such, promoting macrophage polarization from M1 to M2 phenotypes might only be beneficial at a specific phase. To guarantee a more effective outcome for promoting wound repair, temporally programmed therapeutic strategies should still be developed.

### Repairing the tissue

Starting around day 3 and lasting for up to 2 weeks, the proliferation phase follows three sequential processes, including angiogenesis, fibroplasia, and re-epithelialization, focusing on filling the wound defect and restoring the skin barrier. Typically, angiogenesis involves the formation of new blood vessels from the pre-existing ones, supplying oxygen and nutrients to the metabolically active healing tissue^[31,32]^. Then the fibroblasts migrate into the wound bed to synthesize collagen and other extracellular matrix (ECM) components, forming the granulation tissue. Simultaneously, keratinocytes at the edges of the wound proliferate and migrate across the granulation tissue for re-epithelialization^[33]^. Myofibroblasts also begin to contract the wound, pulling the edges of the wound inward to close the wound and reduce the defect^[34]^.

Inheriting abundant bioactive molecules from their parent *Lactobacillus* strain, the L-EVs have been increasingly reported to target endothelial cells, fibroblasts, as well as keratinocytes to promote tissue repair and rebuilding. For instance, the *Lactobacillus rhamnosus GG*-derived EVs have been found enriched in miR-21-5p, which enhances the proliferation and migration capacities of endothelial cells and subsequently promotes angiogenesis, thereby accelerating cutaneous wound healing^[35]^. While miR-21-5p was identified as an essential functional molecule in the presented study, other bioactive cargoes, such as proteins and lipids, have not yet been systematically examined for their contributions to wound healing, particularly through the cooperative regulatory networks with miR-21-5p. The *Lactobacillus plantarum-derived* EVs could reverse ultraviolet (UV)-induced photoaging-related gene expression, enhance proliferation and migration of fibroblasts, and upregulate collagen and elastin synthesis, thereby promoting cutaneous wound repair and skin rejuvenation^[36]^. The *Lactobacillus reuteri-derived* EVs have been characterized with intrinsic miR-21a-5p to enhance the function of keratinocytes and improve epidermal thickness, thereby promoting cutaneous wound healing^[37]^. However, in most current studies, the administration route of these L-EVs remains subcutaneous, which may not be applicable to clinical settings. Advanced delivery systems for the direct topical administration of these L-EVs should be developed in the future^[38]^.

### Remodeling the matrix

The collagen fibers deposited at the wounds during the proliferation phase are usually the disorganized type III collagen. Only until the final remodeling phase of wound healing are these collagen fibers gradually replaced by the organized type I collagen, regaining the integrity and strengthening the tensile strength of the regenerated tissues^[32]^. Although the myofibroblasts are essential for wound contraction, their persistent activity always leads to excessive collagen deposition, scar formation, and even fibrosis^[39]^. Therefore, the quality of the repaired wounds can be controlled by either modulating myofibroblast activity or driving the transition to the remodeling phase.

According to the most recent studies, L-EVs have shown promising anti-fibrotic potential that favors more natural wound regeneration. For instance, the *Lactobacillus casei-derived EVs-loaded* hydrogel significantly reduced the percentage of non-structured epidermis and dermis thickness within the wound site, decreasing scar formation^[38]^. Besides, *Lactobacillus druckerii*-derived EVs inhibited the expression of collagen I/III and α-SMA and cell proliferation of fibroblasts derived from hypertrophic scars, and *in vivo* inhibited the formation of hypertrophic scars in the scleroderma mouse model^[40]^. However, the absence of long-term follow-up for wound remodeling and scar maturation in these studies precluded the evaluation of the sustained anti-scarring and long-term reparative efficacy of L-EVs.

## WHERE THE LACTOBACILLUS DERIVED EVS HEAD FOR FUTURE THERAPY

Despite growing research interest in L-EVs and compelling preclinical evidence of their wound-treatment capacities, several hurdles remain to be tackled for their successful clinical translation. First of all, the isolation and purification standardizations for the L-EVs are lacking. Even though the Minimum Information for Studies of Extracellular Vesicles (MISEV) recommends differential centrifugation at 10,000 × *g* to 20,000 × *g* to collect the EVs, it is only applicable for research purposes^[41]^. As for the industrial-scale manufacturing of the L-EVs for clinical use, the standardized isolation and purification workflow still needs to be established with defined techniques and strict protocols. Secondly, cargo and batch variabilities for the L-EVs. Even the same *Lactobacillus* cultured under different conditions could secrete L-EVs with different structures and compositions^[42]^. These heterogeneities of L-EVs seriously compromise their consistent therapeutic outcomes. Therefore, the international standard quality system remains in high demand to guarantee the precise therapeutic performance of L-EVs. Several critical quality control parameters, such as subtype identity, size, concentration, purity, and batch-to-batch consistency, should be defined to ensure an acceptable range of standards. Thirdly, the classification and regulation of L-EVs are ambiguous. It remains unclear whether the L-EVs-based therapies would be classified as biological products or complex drug delivery systems, which require different chemistry, manufacturing, and controls documentation^[43]^. The specific guidelines for L-EVs-based therapies are still pending issuance by regulatory bodies, such as the Food and Drug Administration (FDA) or the European Medicines Agency (EMA). Last but not least, the clinical studies on L-EVs are currently lacking. Critical data on their safety, immunogenicity, biodistribution, and pharmacokinetic profiles in humans are currently unavailable. Currently, only the live probiotics are commercially available in the food and pharmaceutical industries, whereas the L-EVs are not yet^[44,45]^. Although L-EVs are theoretically expected to possess an improved safety profile compared to live probiotics due to their non-replicative nature, their safety and immunogenicity still require more rigorous and comprehensive assessments to ensure their applicability to immunocompromised or immunodeficient populations.

Beyond the current investigation into their wound-healing mechanisms, future research on L-EVs should focus on their intelligent generation for optimal clinical efficiency. Leveraging artificial intelligence (AI) and multi-omics analyses, a specialized database of L-EVs that is similar to Vesiclepedia, ExoCarta, and EVAtlas of exosomes should be established in the future. It could guide clinicians in quickly prescribing L-EVs for the individual patient with a unique wound environment and metabolic profile, moving toward precision medicine for wound management^[46]^. For instance, Chouaib and colleagues have developed an Intelligent Vesicle Exocytosis Analysis (IVEA) platform, which could be extended to enable multi-label vesicle tracking and in-depth mechanistic quantification in wound therapeutics. The IVEA platform broadly supports diverse fluorescent labels for simultaneous multi-tracer tracking, while automatically extracting comprehensive kinetic parameters, such as fusion kinetics, diffusion radius, and secretion profiles, directly linking EV dynamics to specific wound repair processes like angiogenesis, collagen deposition, and macrophage polarization^[47]^. Besides, the diverse engineering approaches should be exploited in combination to generate L-EVs with improved properties. For instance, the L-EVs can be biologically engineered as EcN 1917 to encapsulate the VEGF^[31]^ and then further chemically engineered to display the targeting ligand on human umbilical vein endothelial cells‌ (HUVECs). As such, the engineered L-EVs could be endowed with endothelium-targeting and angiogenesis-promoting capacities simultaneously to achieve enhanced wound-healing efficacy. Moreover, since topical administration remains the primary route of medication for wound treatment, materials engineering approaches should be proposed and explored to generate advanced L-EVs formulations such as sprayable hydrogels and microneedle patches^[48,49]^. These advanced formulations could not only facilitate the controlled and sustained release of L-EVs around the wound but also improve treatment compliance of patients. Integration of AI technologies with L-EVs engineering can enable the circumvention of translational barriers in L-EVs-based wound treatment, including loading efficiency, targeted delivery, *in vivo* stability and pharmacokinetics, as well as immunogenicity and safety^[50]^. As future research continues to integrate these interdisciplinary technologies, the L-EVs are poised to become the cornerstone of the next generation of wound management modalities, exerting satisfactory clinical outcomes [Figure 3].

**Figure 3:**
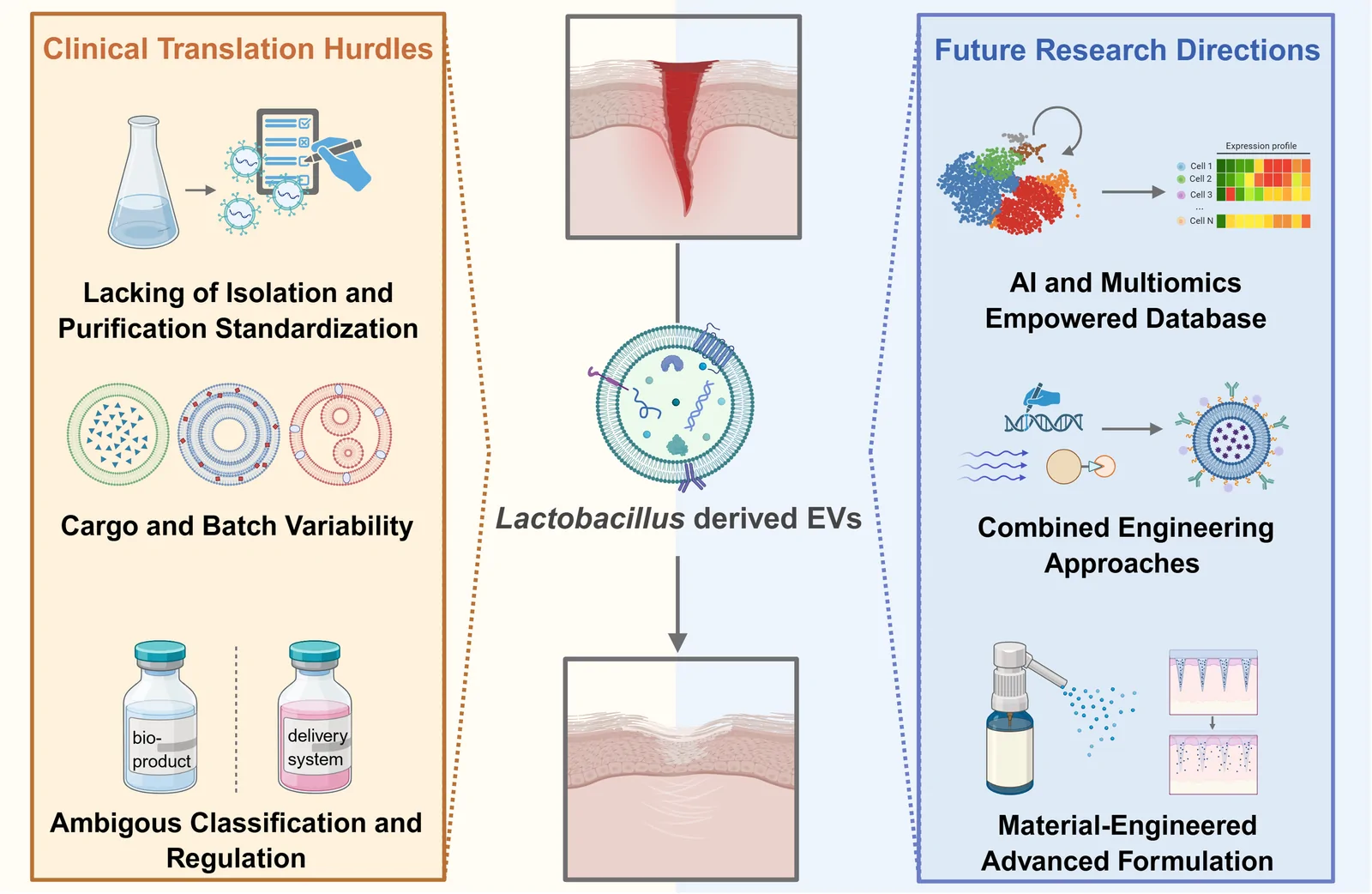
The clinical translation hurdles and future research directions of L-EVs for wound management. Created in BioRender. Shi, Y. (2026). AI: Artificial intelligence; EV: extracellular vesicle; L-EV: *Lactobacillus*-derived extracellular vesicle; multiomics: multi-omics.

## DECLARATIONS

### Copyright

© The Author(s) 2026.
